# Resilience in the face of adversity: classes of positive adaptation in trauma-exposed children and adolescents in residential care

**DOI:** 10.1186/s40359-023-01049-x

**Published:** 2023-01-30

**Authors:** Katharina Sölva, Alexander Haselgruber, Brigitte Lueger-Schuster

**Affiliations:** Unit of Psychotraumatology, Department of Clinical and Health Psychology, Faculty of Psychology, University of Vienna, Wächtergasse 1, 1010 Vienna, Austria

**Keywords:** Resilience, Childhood trauma, Residential care, Protective factors, Latent class analysis

## Abstract

Children and adolescents in residential care often face multiple traumatic experiences. However, some individuals show resilient adaptation. To depict this heterogeneity, the person-centered examination of different classes of adaptation is a powerful tool. Up to date, resilience was insufficiently addressed in this population. Data from 141 children and adolescents in residential care institutions in Austria regarding trauma history, psychopathology, behavioral adjustment, and protective factors were assessed with standardized self-report questionnaires. Distinct classes of adaptation after traumatic experiences were examined with Latent Class Analysis. Class differences regarding traumatic experiences and protective factors were analyzed with χ^2^ testing. Three classes were identified [resilience (66.18%), mixed psychopathology (13.97%, mixed), high psychopathology (19.85%, high)]. Only males were classified into the resilient class and only females into the high class. The high class differed significantly from the resilient class regarding cumulative trauma history and protective factors. The mixed class did not differ from the resilient class regarding trauma history, however, they differed significantly regarding protective factors. The resilient class was associated with protective factors. Strong gender differences show the relevance of a differentiated evaluation of gender-specific protective factors and resilience indicators. Fostering protective factors may be a suitable approach for tailored intervention measures.

**Supplementary Information:**

The online version contains supplementary material available at 10.1186/s40359-023-01049-x.

## Background

### Resilience

With the growing awareness of the multitude of traumatic experiences faced by children and adolescents, research on resilience is gaining importance [1]. Resilience is defined as “the capacity of a system to adapt successfully to challenges that threaten the function, survival, or future development of the system (p. 2)” [1] and considered a common phenomenon arising from normative processes, which each individual encounters during development [2, 3]. Resilience is a dynamic process, arising from the interplay between risk and protective factors [4]. This research aims to examine the complex and dynamic processes of positive adaptation in the aftermath of traumatic experiences [1]. There is a broad consensus that resilience is only present if, in addition to adaptation, a certain degree of risk is discernible [5]. According to Masten [4] three elements ought to be addressed to investigate resilience, namely (1) risk factors, (2) indicators of adaptation and (3) influences that foster resilience, commonly referred to as protective factors. Despite a broad consensus in regard to the main characteristics of resilience, there is no clear definition of the concept and consequently no agreement in regard to which indicators should be included in the assessment of resilience [1].

#### Risk factors

One primary goal of resilience research is the identification of risk factors that threaten the balance of a system [1, 6]. In the context of childhood risk factors, a range of different experiences have been linked to negative outcomes. One risk factor that has often been associated with complex post-traumatic symptoms is childhood maltreatment, including different types of abuse and neglect. Early maltreatment is especially associated with disturbances in self-organization (DSO) [7] and a wide range of psychopathological problems [8]. Other traumatic experiences like natural disasters [9, 10], accidents [11], sudden death of a close family member [12], and different types of violence in the family or community [13] have also been linked to mental health problems and psychopathology in childhood and adolescence [for reviews see [14–16]. Traumatic experiences are often cumulative, referring to multiple different subtypes of trauma being experienced by the same individual [1, 17, 18].

#### Indicators of adaptation

Another main goal of resilience research is the examination of an individual’s response to risk factors [1, 6]. It has been shown that there is a great heterogeneity in the sequelae of traumatic experiences, leading to a wide range of different outcomes [19]. Frequently reported consequences of traumatic experiences among children and adolescents are posttraumatic stress disorder (PTSD) and complex PTSD (CPTSD) [20], dissociative symptoms [21], internalizing and externalizing behavioral problems [22], and a range of other adjustment problems such as interpersonal problems [23] and sleeping and thought problems [24, 25]. However, some individuals show resilience despite traumatic experiences [6, 26, 27].

There are shortcomings in previous research. First, resilience was often examined with variable-centered methods, which are not able to detect the post-traumatic heterogeneity and different subgroups of adaptation [28, 29], because this approach describes relationships at variable level. The variable-centered approach is based on the assumption that the members of a group are homogeneous and consequently certain processes and outcomes within the group are similar [30]. Another shortcoming is the examination of factors as indicators of resilience, which conceptually are defined as protective factors and not as indicators of resilience, for example self-esteem [26]. Furthermore, some relevant consequences of traumatic experiences such as CPTSD have not been examined sufficiently in the population of children and adolescents in residential care [29].

#### Protective factors

A third goal of resilience research is the identification of protective factors that are associated with positive adaptation in the context of risk [2, 31]**.** Through a profound understanding of those factors, it may be possible to promote resilience in children and adolescents at risk [32]. Protective factors are often categorized into assets and resources. Assets refer to characteristics of an individual, while resources describe external support available to the individual [33].

##### Assets

Several individual characteristics have been linked to positive adaptation in the aftermath of traumatic experiences. The assets self-efficacy and sense of coherence (SOC) have shown to be particularly important for positive adaptation [3, 34, 35]. Self-efficacy was able to distinguish maladaptive from resilient youths, which showed positive adaptation in three domains (rule-abiding conduct, academic achievement, social competence) [36]. Furthermore, self-efficacy was positively associated with resilient outcomes in youth in foster care [37] and adolescents in the general population in Italy [38]. SOC has been found to moderate the relationship between traumatic experiences and the complex post-traumatic symptoms of disturbances in self-regulation (DSO) [39] and furthermore, it has been shown to moderate between experienced violence and reactions associated with stress in adolescents [40, 41].

##### Resources

One of the main external factors that has often been linked to resilience after traumatic experiences are supporting relationships with caregivers and peers [32, 42, 43]. For example, those kinds of relationships were associated with resilient adaptation and fewer risk behaviors in youth exposed to violence [44, 45] and in adolescent girls in foster care after sexual abuse [46]. The overall constellation of several different assets and resources is considered more influential on positive adjustment after trauma than the presence of one certain factor [26, 47].

According to the focus of the current study, singular factors are not discussed in detail here, as this would go beyond of the scope of this study. For reviews, see [32, 34, 35, 42].

### Children and adolescents in residential care

It is relatively common for children and adolescents in residential care to have suffered from a wide range of traumatic experiences [48]. Despite the relatively high level of exposure to risk factors in this population, approximately half of children and adolescents showed resilience in one or more domains of psychopathology or in different domains of functioning across several studies [29, 49, 50]. Usually, high-risk populations like children in residential care are considered to be resilient if they do not show any significant psychopathological or behavioral impairment [6]. Regardless of the vulnerability of children and adolescents in residential care, to this date, patterns of adaption following traumatic experiences among this population have not been sufficiently examined [29]. One reason for this is that it is difficult for traditional, variable-centered statistical methods to depict the heterogeneity after traumatic experiences [28].

### Heterogeneity in post-traumatic sequelae: classes of positive adaptation

Person-centered approaches like the Latent Class Analysis (LCA) are suited to reflect the heterogeneity after traumatic experiences, as they are able to identify latent classes of individuals who show similar response patterns to categorical data [30, 51]. Person-centered approaches can contribute to a better understanding of the variety and heterogeneity of psychological outcomes of children and adolescents in residential care [52]. So far, this population has not been sufficiently examined with person-centered methods regarding adaptation after traumatic experiences. Four studies on children and adolescents in residential care found three [29], and four classes of adaptation [50, 53, 54], respectively. However, the studies differed considerably in the included indicators of adaptation. In the study by Gallitto et al. [29], the following six areas of psychopathology were examined: Anxiety, depression, post-traumatic stress, dissociation, anger, and sexual concerns. Yates and Grey [50] included a wide array of indicators in different areas, including competence (educational, relational, occupational, civic engagement), adjustment (socioemotional and behavioral), self-esteem and psychopathology. The studies by Miller et al. [54] and Keller et al. [53] examined youth in transition to adulthood and only included indicators of external adjustment (education and employment, problem behaviors and current living situation). Studies in other vulnerable adolescent populations found four [55] or three [56] classes. The first study used indicators of psychopathology (depression and post-traumatic symptoms), whereas the latter used a mixed set of indicators of resilience, spanning many different domains (self-esteem, coping, academic efficacy, peer relations, parental discipline, parental supervision, school commitment, daily hassles, neighborhood cohesion, support, attitude towards substances, substance use, and delinquency).

Common to all studies was the identification of one class showing resilience/minimal psychopathology and at least one class showing maladaptation/severe psychopathology. These studies did, however, not sufficiently examine risk factors, which are a crucial factor in the assessment of resilience. Furthermore, the studies differed considerably regarding the included indicators of resilience and adaptation. Up to date, no study included protective factors and their association with classes of adaptation.

Since research has demonstrated strong differences between males and females both in psychopathology and in the incidence and patterns of traumatic experiences [57], gender should be taken into consideration. However, data on gender differences are highly inconsistent. While some studies focusing on resilience report better adaptation in females [58, 59], other studies concerning negative consequences of traumatic experiences have shown that females exhibit a higher probability of reporting post-traumatic stress symptoms, dissociative symptoms [25, 60], lower quality of life [61], depressive symptoms [25, 62], and internalizing problems [48] than males. These inconsistencies may be attributable to differences in the focus of the studies on either resilience or negative consequences of traumatic experiences.

### The present study

The overall aim of the present study is a comprehensive investigation the three factors included in resilience research, namely risk factors, indicators of adaptation and protective factors. For this purpose, risk factors in terms of interpersonal maltreatment (physical, sexual and emotional abuse and physical and emotional neglect) and other potentially traumatic experiences like the experience of an accident, natural disaster, sudden death of a family member, and experiencing violence outside the family were assessed. Furthermore, the frequently associated post-traumatic sequelae dissociative symptoms, internalizing and externalizing behavioral problems, interpersonal problems, thought problems and the ICD-11 posttraumatic symptom clusters PTSD and DSO were included as indicators of resilience, respectively its absence. Additionally, the protective factors self-efficacy, SOC, peer support and caregiver support were included. This broad variety of factors were included to gain a comprehensive picture of the vulnerable population of children and adolescents in residential care.

- The first aim of the study was the examination of different classes of adaptation after traumatic experiences of children and adolescents in residential care in Austria.
- The second aim was the examination of the relationship between classes of adaptation and the included protective factors to distinguish poorly from well-adapted individuals.

## Method

### Participants

Participants were children and adolescents currently living in six different government-run residential care institutions in Lower Austria. The participants were selected based on the following inclusion criteria: Sufficient German language skills, age from 10 to 18 years and the experience of at least one potentially traumatic event. Participation in the study was not possible in the case of a current drug addiction, suicidality, psychotic symptoms, or severe intellectual impairment to the extent that participation was not possible. Initially, 157 participants met the criteria and were included in the study. Sixteen participants were excluded because the questionnaires were not processed properly due to cognitive difficulties or unwillingness. This resulted in a final sample of 141 participants. The final sample consisted of 30.5% female and 69.5% male participants aged 10 to 18 (*M* = 14.43, *SD* = 2.45).

### Measures

#### Traumatic experiences

For the assessment of traumatic experiences, two different questionnaires were used. The Childhood Trauma Questionnaire (CTQ) [63] was used to assess five different types of childhood maltreatment, which comprise physical, sexual and emotional abuse and physical and emotional neglect. Each scale is composed of five items, rated on a five-point Likert scale (‘*Never true’* to ‘*Very often true’*)*.* Overall, the internal reliability for the abuse and neglect items was good: Emotional abuse (Ω = 0.87), physical abuse (Ω = 0.86), sexual abuse (Ω = 0.90), emotional neglect (Ω = 0.89), with one exception, namely physical neglect (Ω = 0.46). The low reliability is in line with previous studies [64, 65]. For the present analysis, the CTQ subscales were used in two ways. First, they were interpreted dichotomously to build a sum score for overall cumulative traumatic experiences and second, the sum score was calculated for each subscale to examine differences in the classes regarding childhood maltreatment thoroughly.

The Child and Adolescent Trauma Screen (CATS) [66] was used to dichotomously (*‘Yes’/’No’*) assess 15 potentially traumatic events. This questionnaire enquires about the experience of physical and sexual abuse, a natural disaster, serious accident or injury, robbery, the sudden or violent death of a loved one, distressing medical treatment and it assesses if the participants were beaten (within or outside the family) or seen someone being beaten (within or outside the family), assaulted with a knife or firearms or witnessed an attack with a knife or firearms and if they have been in a war zone, or if they experienced any other traumatic event. For the current analysis, a cumulative score of experiences was calculated. The experience of physical (three items) and sexual abuse (two items) was not included in this score, as they were previously assessed by the CTQ. The combination of the two questionnaires resulted in a cumulative trauma score ranging from 0 to 15.

#### ICD-11 PTSD and CPTSD

The ICD-11 symptom clusters PTSD and DSO were measured with the International Trauma Questionnaire- Child and Adolescent Version (ITQ-CA) [67]. This questionnaire assesses symptoms of PTSD and CPTSD according to the ICD-11 [68] and was recently translated and validated in German [20]. It consists of six items capturing PTSD and six items that cover DSO. Together, DSO and PTSD constitute CPTSD. The items are rated on a five-point Likert scale (*‘Never’* to *‘Almost always’)*. Dichotomous values were calculated based on the cut-offs. In this study, the scales showed an acceptable through good Omega Ω of 0.074 (PTSD) and 0.87(DSO).

#### Behavioral adjustment

To measure internalizing and externalizing behavioral adjustment, the Child Behavior Checklist, Youth Self Report (CBCL, YSR 11-18R) [69] was used. This questionnaire assesses different behavioral problems with 113 Items rated on a three-point Likert scale (‘*Never*’ to ‘*Always’).* Internalizing problems comprise symptoms of depression, anxiety and somatic symptoms, externalizing problems comprise aggressive and rule-breaking behavior. Furthermore, the additional scales interpersonal problems and thought problems were included. Dichotomous values of the individual scales were used for the calculation. These were formed on the basis of the reported cut-offs from the manual [69]. For the different subscales, good through excellent internal consistencies were found. The analyses showed a Cronbach’s *α* for interpersonal problems (Ω = 0.73), thought problems (Ω = 0.81), internalizing problems (Ω = 0.89), externalizing problems (Ω = 0.87).

#### Dissociation

To assess dissociation, the Adolescent Dissociative Experiences Scale-8 (ADES-8) [70] was used. This questionnaire consists of eight items on a numerical scale ranging from one to ten. The dichotomous score (significant/non-significant symptoms) was used for the calculations. The cut-off score was based on the manual. Internal consistency for this scale was good (Ω = 0.83).

#### Protective factors

Protective factors were assessed with the Questionnaire for Resources in Children and Adolescents (FRKJ, Fragebogen für Ressourcen im Kindes- und Jugendalter) [71]. This questionnaire consists of 60 items, which are rated on a four-point Likert scale (‘*Never true*’ to ‘*Always true*’). The factors self-efficacy, SOC, peer support, and caregiver social and emotional support were considered. The subscales showed an overall good through excellent internal consistency with an Omega Ω for self-efficacy (Ω = 0.91), SOC (Ω = 0.82), peer support (Ω = 0.87), and caregiver social and emotional support (Ω = 0.88). For a detailed description of the cut-off scores for the individual measures, see Additional file 1.

### Procedure

The present data were gathered during a large research project which was authorized by the government of Lower Austria and carried out longitudinally. Within the first measurement period (May–October 2018), childhood maltreatment in the family of origin was assessed with the CTQ. Within the second measurement period (March–September 2019), data on other potentially traumatic experiences (CATS), psychopathological symptoms (ITQ-CA, ADES-8), behavioral adjustment (CBCL- YSR), and protective factors (FRKJ) were collected. Ethical approval was granted beforehand by the University of [blinded for review] (#00328). Participants and their caregivers provided written consent before the beginning of the study. The survey was conducted in the institutions. Data collection was carried out using self-report questionnaires, whereas two participants were being supported by a clinical psychologist or a master student under supervision. The participants could ask questions at any time. Where any potential difficulties of the participant (motivation, concentration, reading abilities) were known, an individual setting was provided, where one participant was supported by one clinical psychologist or a master student.

### Missing data

Considering the indicators for resilience, the following percentages of missing data were observed: PTSD (2.1%) and DSO (2.8%), dissociative symptoms (10.6%), interpersonal problems (1.4%), thought problems (2.1%), internalizing problems (2.1%) and externalizing problems (2.8%). Considering protective factors, the following percentages of missing data were observed: Self-efficacy (10.6%), SOC (9.9%), peer support (21.1%), and caregiver support (13.5%). For the traumatic experiences, missing values between 0.7 and 2.1% were observed. Based on a non-significant result in the Little’s MCAR test (χ^2^(19) = 15.30, *p* = 704), it can be concluded that the data are missing completely at random (MCAR). Missing data was handled with full information maximum likelihood [72].

### Data analysis

Data was analyzed using IBM SPSS Statistics 25 and Mplus 7.0 [73]. LCA and group comparisons were performed using Mplus 7.0. For descriptive statistics and ANCOVA, IBM SPSS Statistics 25 was used. LCA was used to determine the number of latent classes that best represented the data. Psychopathology as well as behavioral adjustment were used as dichotomous indicators. The number of starts and iterations was set at 5000 and 1000, respectively, to avoid potential issues with local maxima [74]. To identify the best class solution, the fit indices of successively calculated models (2–5 classes) were compared. An entropy value < 0.80 indicates an unreliable classification of respondents [74]. The fit indices AIC [75], BIC [76] Klicken oder tippen Sie hier, um Text einzugeben.and SABIC [77] were included in the decision. In all cases, the model with the lowest values across the different fit indices is considered the best solution. Non-significant values of the Bootstrap Likelihood Ratio Test (BLRT) indicate that k-1 classes are a better fit [78]. Usually, the fit indices are not clearly interpretable and therefore, all indices as well as interpretability should be considered when deciding on the optimal number of classes.

To examine the association between latent classes, protective factors, and traumatic experiences, the three-step method was implemented in Mplus [79]. This method can account for classification error by estimating the latent classes, fixing the parameters, and including auxiliary variables [78]. With the three-step method for distal outcomes (DE3STEP), χ^2^ tests can be calculated to examine the differences between the groups considering the different protective factors and traumatic experiences. Considering differences in traumatic experiences was especially crucial to determine whether participants of emerging classes could in fact be described as resilient as opposed to just having experienced significantly fewer traumatic events. Gender and cumulative traumatic experiences were included as covariates in a follow-up ANCOVA.

## Results

### Descriptive statistics

Means and standard deviations for traumatic experiences, indicators of adaptation, and protective factors for the overall sample are presented in Table 1. Comparison of means and standard deviation for females and males regarding traumatic experiences, indicators of adaptation, and protective factors are presented in Table 2. The number of different traumatic experiences reported by the participants ranged from 1 to 13 (*M* = 5.58, *SD* = 2.25). Regarding sociodemographic data, most of the participants attended a special needs school (48.2%), followed by secondary school (28.2%) and vocational training school (20.0%) at the time of the survey. Most children and adolescents had regular contact with their parents (96.2%). Among those, 87.0% met their parents once a week or more often, 11.4% once or several times per month.

**Table 1:** Descriptive statistics of traumatic experiences, indicators of adaptation, and protective factors

	*M (SD)*	Range
*Traumatic experiences*		
Emotional abuse	9.01 (4.82)	0–25
Physical abuse	7.36 (4.32)	0–25
Sexual abuse	6.07 (3.09)	0–25
Emotional neglect	16.79 (6.07)	0–25
Physical neglect	12.02 (3.32)	0–25
CATS cumulative	3.04 (1.81)	0–10
Cumulative	5.58 (2.25)	0–15
*Indicators of adaptation*		
PTSD	6.08 (4.91)	0–30
DSO	6.26 (5.87)	0–30
Dissociation	1.67 (1.86)	0–10
Internalizing problems	14.90 (11.63)	0–62
Externalizing problems	14.01 (9.48)	0–64
Interpersonal problems	4.47 (3.44)	0–22
Thought problems	6.09 (4.74)	0–24
*Protective factors*		
Self-efficacy	16.92 (4.66)	6–24
SOC	17.49 (4.12)	6–24
Caregiver support	17.91 (5.00)	6–24
Peer support	19.18 (4.33)	6–24

*M* mean, *SD* standard deviation, *Range* possible range of the scale, raw data, *CATS cumulative* cumulative score of potentially traumatic life events according to the CATS, *cumulative* total cumulative score of CTQ and CATS combined

**Table 2:** Comparison of means and standard deviation for females and males regarding traumatic experiences, indicators of adaptation and protective factors

	Female	Male	t(df)	*p*	95% CI		*d*
	*M (SD)*	*M (SD)*			Lower	Upper	
*Traumatic experiences*							
Emotional abuse	11.35 (6.01)	7.96 (3.77)	− 3.41 (57.33)	0.001*	− 5.38	− 1.40	0.7
Physical abuse	8.05 (4.87)	7.04 (4.03)	− 1.27 (136)	0.207	− 2.57	0.56	0.2
Sexual abuse	7.60 (4.62)	5.37 (1.67)	− 3.08 (47.04)	0.003*	− 3.70	− 0.78	0.6
Emotional neglect	16.14 (5.54)	17.08 (6.30)	0.85 (137)	0.399	− 1.26	3.15	0.2
Physical neglect	11.84 (3.29)	12.10 (3.34)	0.44 (137)	0.663	− 0.94	1.47	0.1
CATS cumulative	3.23 (1.69)	2.97 (1.86)	− 0.75 (133)	0.453	− 0.93	0.42	0.1
Cumulative	6.33 (2.08)	5.26 (2.25)	− 2.45 (116)	0.016*	− 1.95	0.21	0.5
*Indicators of adaptation*							
PTSD	9.33 (4.80)	4.71 (4.28)	− 5.46 (130)	< 0.001**	− 6.30	− 2.95	0.9
DSO	10.27 (6.36)	4.55 (4.73)	− 5.17 (59.72)	< 0.001**	− 7.93	− 3.51	1.0
Dissociation	2.49 (2.36)	1.34 (1.50)	− 2.75 (48.58)	0.008*	− 1.99	− 0.31	1.0
Internalizing problems	21.78 (12.29)	11.99 (10.07)	− 4.50 (63.81)	< 0.001**	− 14.14	− 5.45	0.9
Externalizing problems	16.05 (9.37)	13.14 (9.44)	− 1.66 (135)	0.100	− 6.39	0.56	0.3
Interpersonal problems	5.45 (3.70)	4.05 (3.25)	− 2.23 (137)	0.027*	− 2.64	− 0.16	0.4
Thought problems	8.00 (4.74)	5.28 (4.53)	− 3.18 (136)	0.002*	− 4.41	− 1.03	0.6
*Protective factors*							
Self-efficacy	15.22 (4.70)	17.63 (4.48)	2.17 (124)	0.008*	0.66	4.17	0.5
SOC	15.63 (4.23)	18.28 (3.83)	3.46 (125)	0.001*	1.13	4.17	0.6
Caregiver support	16.86 (5.21)	18.36 (4.88)	1.52 (120)	0.132	− 0.46	3.46	0.3
Peer support	17.43 (4.99)	19.92 (3.81)	3.02 (122)	0.003*	0.86	4.12	0.6

*M* mean, *SD* standard deviation, *95% CI* 95% Confidence interval, *lower* lower boundary, *upper* upper boundary, *d* Cohens *d*, *CATS cumulative* cumulative score of potentially traumatic life events according to the CATS, *cumulative* total cumulative score of CTQ and CATS combined, *significant values p < .05, ** significant values p < .001

### Latent class analysis

According to the results of the LCA, a 3-class model was deemed the optimal solution. For the fit indices, see Table 3.

**Table 3:** Model fit indices of latent classes of adaptation after traumatic experiences

C	LL	AIC	BIC	SABIC	Entropy	BLRT *p*
2	− 384.992	799.984	**843.674**	796.222	0.909	− 461.286 < 0.001
3	− **374.442**	**794.885**	861.876	**789.116**	**0.816**	− **384.992** **0.0200**
4	− 368.855	799.710	890.002	791.935	0.859	− 374.442 0.6667
5	− 363.535	805.070	918.663	795.288	0.915	− 368.855 0.3636

*C* number of classes, *LL* log-likelihood, *AIC* Akaike Information Criterion, *BIC* Bayesian Information Criterion, *SABIC* Sample-size adjusted Bayesian Information Criterion, *BLRT* bootstrapped likelihood ratio test, *p p-*value

Bolded values indicate the best fitting parameter for each model

**Fig. 1:**
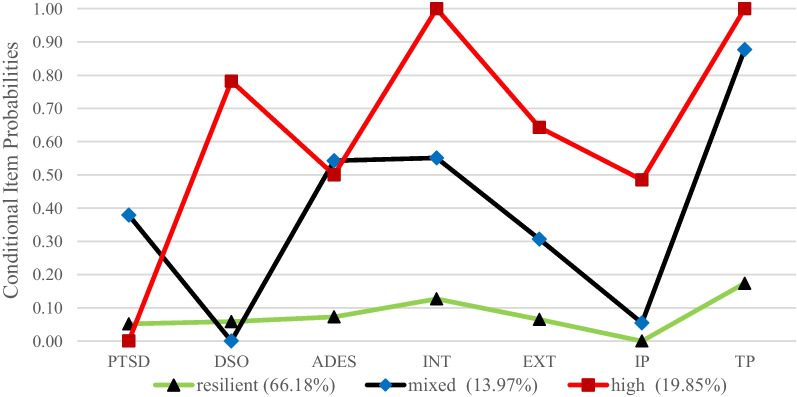
Visual representation of the results of the latent class analysis. Methods: This analysis was conducted with latent class analysis. The figure shows the conditional item probabilities for the seven different indicators regarding the membership to one of the three classes. The indicators were posttraumatic stress disorder (PTSD), disturbances in self-organization (DSO), dissociative symptoms (ADES), internalizing problems (INT), externalizing problems (EXT), interpersonal problems (IP), thought problems (TP). Results: The analysis yielded three different latent classes, which were labelled according to their characteristics on the indicators. The resilient class (green) showed low rates on all indicators, the mixed psychopathology class (blue) showed high rates for dissociative symptoms, internalizing problems, and thought problems**,** moderate rates of externalizing problems and PTSD**,** and low rates of DSO and social problems, and the high psychopathology class (red) showed high rates on all indicators, except PTSD

The three-class solution is based on the lowest values on the AIC and the SABIC, an acceptable Entropy > 0.80 and a significant BLRT. Based on this solution, the individuals could be classified appropriately. The three classes were labelled according to their distinctive response pattern across the indicators. Class 1 was labelled resilient (66.18%, 0% female, *n* = 90), class 2 was labelled mixed psychopathology (13.97%, 57.1% female, *n* = 19), and class 3 was labelled high psychopathology (19.85%, 100% female, *n* = 27). For an illustration of the distinct classes, see Fig. 1.

### Class comparison

The resilient class showed the lowest symptom severity on almost all indicators. The mixed symptomatology class showed mixed patterns on the indicators. It showed the highest rates of dissociative symptoms and PTSD, high rates of thought problems and internalizing problems**,** moderate rates of externalizing problems**,** and low rates of DSO and social problems. The high symptomatology class showed high rates of DSO, internalizing problems, externalizing problems, interpersonal problems, and thought problems, high rates of dissociative symptoms and low rates of PTSD. For a visual representation of the distinct patterns on the indicators for the three classes, see Fig. 1.

The classes did not differ in terms of age, however, they showed significant differences regarding a few distinct traumatic experiences and all protective factors. Furthermore, the classes showed significant differences regarding gender, with only females being in the high symptomatology class and only males being in the resilient class.

Regarding traumatic experiences, the mixed symptomatology class did not differ from the resilient class. Furthermore, the mixed symptomatology class differed from the high symptomatology class only in the experience of emotional abuse. In addition, the resilient class differed from the high symptomatology class only in the experience of emotional and sexual abuse and the cumulative score of all traumatic experiences. For a descriptive comparison and χ^2^ differences of traumatic experiences and protective factors depending on classes of adaptation, see Table 4.

**Table 4:** Descriptive comparison and χ^2^ differences of traumatic experiences and protective factors depending on classes of adaptation

	Classes		Comparison	Classes		Comparison	Classes		Comparison
	Resilient M (SD)	Mixed M (SD)	χ2	Mixed M (SD)	High M (SD)	χ2	Resilient M (SD)	High M (SD)	χ2
*Trauma*									
EA	7.58 (0.35)	8.34 (0.81)	0.81	8.34 (0.81)	14.35 (2.19)	5.25*	7.58 (0.35)	14.35 (2.19)	8.91*
PA	6.62 (0.54)	8.69 (2.75)	0.44	8.69 (2.75)	8.86 (1.49)	0.00	6.62 (0.54)	8.86 (1.49)	2.66
SA	5.30 (0.26)	5.49 (0.34)	0.48	5.49 (0.34)	9.28 (1.74)	3.81	5.30 (0.26)	9.28 (1.74)	4.42*
EN	16.68 (0.70)	18.75 (1.57)	1.32	18.75 (1.57)	15.03 (1.11)	3.36	16.68 (0.70)	15.03 (1.11)	1.56
PN	12.14 (0.39)	12.48 (0.69)	0.16	12.48 (0.69)	11.35 (0.71)	1.18	12.14 (0.39)	11.35 (0.71)	0.97
CATS	3.48 (0.38)	3.45 (0.55)	0.00	3.45 (0.55)	2.73 (0.22)	1.22	3.48 (0.38)	2.73 (0.22)	3.05
Cumulative	4.83 (0.36)	6.93 (2.06)	0.81	6.93 (2.06)	7.12 (0.68)	0.01	4.83 (0.36)	7.12 (0.68)	14.54**
*Protective*									
SE	19.37 (2.22)	13.46 (1.05)	4.97*	13.46 (1.05)	17.15 (0.52)	10.42*	19.37 (2.22)	17.15 (0.52)	0.82
SOC	18.60 (1.02)	13.53 (0.76)	15.16**	13.53 (0.76)	18.38 (0.42)	31.21**	18.60 (1.02)	18.38 (0.42)	0.04
Caregiver	23.34 (0.26)	13.69 (1.01)	87.69**	13.69 (1.01)	18.06 (0.53)	13.78**	23.34 (0.26)	18.06 (0.53)	78.28**
Peer	21.61 (1.61)	15.24 (1.10)	9.80*	15.24 (1.10)	19.87 (0.43)	15.65**	21.61 (1.61)	19.87 (0.43)	0.92
Sum	18.91 (0.78)	12.77 (0.99)	25.83**	12.77 (0.99)	17.56 (0.55)	14.49**	18.91 (0.78)	17.56 (0.55)	1.90

*M* mean, *SD* standard deviation, *mixed* mixed symptomatology, *high* high symptomatology; *EA* emotional abuse, *PA* physical abuse, *SA* sexual abuse, *EN* emotional neglect, *PN* physical neglect, *SE* self-efficacy, *SOC* sense of coherence, *Sum* sum of protective factors, *CATS* cumulative score of potentially traumatic life events according to the CATS, *cumulative* total cumulative score of CTQ and CATS combined

*Significant values *p* < .05; **significant values *p* < .001

Gender and cumulative traumatic experiences were included as covariates in an ANCOVA. There was no significant interaction of gender and cumulative traumatic experiences in the relationship between classes of adaptation and protective factors in three out of four factors (self-efficacy, SOC and peer support). Regarding caregiver support, both gender (*F*(1) = 5.31, *p* = .023) and cumulative traumatic experiences (*F*(1) = 128.44, *p* = .007) were significant covariates. However, the class differences between the latent class regarding caregiver support were still significant after including the covariates (*F*(2) = 14.48, *p* < .001).

## Discussion

In the present study, three distinct classes of children and adolescents in residential care exposed to traumatic events were found. Those classes showed distinctive patterns on indicators of psychopathology and behavioral adjustment, protective factors, and traumatic experiences. These classes represent distinct groups of individuals, adapting differently and distinctively to traumatic experiences. The first class showed resilient adaptation despite traumatic experiences, the second class showed mixed patterns on the indicators and the third class depicted high symptomatology. Surprisingly, the resilient class only comprised male participants and the high symptomatology class only comprised females. The classes differed significantly in all protective factors. However, the classes did not differ significantly in most of the traumatic experiences.

As resilience refers to positive adaptation despite the exposure to adversity [4], different traumatic experiences were examined. All participants stated that they had experienced at least one traumatic event. This is rather common for children and adolescents in residential care [60]. Although the high symptomatology class and the mixed symptomatology class descriptively showed higher scores in almost all subscales of traumatic experiences than the resilient class, there were only few significant differences regarding traumatic experiences between the classes. There was no significant difference between the three classes regarding the experiences of physical abuse, emotional neglect, physical neglect and in the cumulative score of traumatic experiences according to the CATS. Furthermore, there was no significant difference between the resilient and the mixed symptomatology class regarding all traumatic experience. This points towards the importance of a person-centered investigation of resilience, because this distinct group of individuals could be detected that showed positive adaptation despite adversity.

Comparing the overall cumulative score of traumatic experiences, significant differences between the resilient and the high symptomatology class were found, with the high symptomatology class reporting significantly more traumatic experiences. These results are in agreement with data obtained in prior studies, showing that the probability of an unfavorable adaptation increases with the number of stressors [17, 18]. Interestingly, the mixed symptomatology class did not differ significantly from the resilient class in any of the traumatic experiences. This result corroborates the findings from other studies, which report resilient adaptation despite traumatic experiences [49, 80]. This may indicate that these classes do not differ because of their traumatic experiences, but presumably because of other factors related to resilience such as protective factors. Self-esteem, SOC, and supporting relationships have been shown to foster resilient adaptation in the context of adversity [3, 26].

The finding regarding the number of classes partly confirms earlier studies examining children and adolescents in residential care [29] and adolescents in public housing [56], which also found three classes of adaptation after traumatic experiences. In contrast, four classes were identified in populations of emancipated foster youth [50], foster youth in transition to adulthood [54, 55] and female sexual assault survivors [55]. Up to date, only few studies used person-centered methods to investigate resilience. Only one study assessed resilience in foster youth with a person-centered approach [50]. Hereby, a comprehensive selection of resilience indicators was examined, including different areas of psychopathology, competence, and adjustment. The analyses resulted in four classes, differing the present study. However, as the previous study assessed markedly diverse indicators of resilience, including protective factors like self-esteem [50], this may have influenced the number of classes. Two further studies assessed adaptation in foster youth in transition to adulthood [54, 55]. However, those studies only included external indicators for adaptation, which may not reflect resilience in this population.

Regarding the characteristics of the distinct classes, the largest class of children and adolescents in residential care could be classified as resilient based on different indicators of psychopathology and behavioral adjustment. This class showed low symptom severity on all the indicators, which is in line with previous studies examining adaptation after traumatic experience in a population of children and adolescents in residential care [29, 50]. The second largest class depicted high psychopathology. This can be compared to studies examining resilience, where at least several of the participants under investigation showed high psychopathology/maladaptation [49, 50, 80]. The smallest class showed mixed psychopathology. This class showed very high and very low symptom severity across the different indicators. This class can be compared to an ‘externally resilient’ class in another study [50]. Like the mixed psychopathology class in the present study, the ‘externally resilient’ class showed low externalizing problems and high internalizing problems [50]. This indicates that, although this class is externally adapted and appears to be coping well on the outside, it exhibits a high level of psychopathological problems.

One very prominent finding are the gender differences between the classes. The resilient class comprised only males and the high symptomatology class comprised only females. On one hand, this result is in line with previous studies examining psychopathology after traumatic experiences, which found that females show overall more and also different psychopathological symptoms [48, 60, 62]. However, this contrasts studies examining resilience, which found females to be more likely to show resilient outcomes [58, 59]. However, in the studies examining resilience, mostly externalizing behavior [58] and indicators associated with problems in interpersonal relationships and empathy were assessed [59]. Since females are less likely to exhibit externalizing symptoms [48] and are more likely to engage in interpersonal relationships [81], this could explain the gender differences between the studies. Furthermore, the observed gender differences could be attributed to the low levels of interpersonal resources reported by females in the present study. Previous studies found that females are more likely to benefit from supportive relationships than males [81] and the lack of social support in the present study could have affected psychopathological symptoms in females. In addition, males may not have been able to identify and report traumatic experiences and related symptoms and may therefore seem resilient. These results match those observed in an earlier study, where females were able to identify trauma-related symptoms and anxiety better than males [82].

Consistent with the literature, the resilient class showed the highest values for all protective factors. This further supports theories of positive adaptation, which assume that the availability of different protective factors to an individual contributes to positive adaptation and well-being [83]. In turn, this usually increases other protective factors because assets and resources are hypothesized to influence each other [84]. Through this mechanisms, upward spirals of protective factor acquisition are enabled, which may have affected the adaptation [85].

The values for the different protective factors were lowest in the mixed symptomatology class. This class differed significantly from both other classes regarding protective factors. One possible explanation for this are the remarkably high scores in problems associated with internalizing problems in the mixed symptomatology class, which are often associated with a poor self-image [86] and diminished perceived peer-support [87]. Combined with the high PTSD symptoms, which were shown to be negatively associated with supporting relationships [88] and self-esteem [89], this could explain the low scores regarding protective factors in the mixed symptomatology class.

### Limitations

The present study is limited by the assessment of potentially traumatic experiences at two different measurement periods. In the first period, maltreatment within the family prior to residential care placement was assessed and in the second period, other traumatic experiences were assessed. Furthermore, self-report questionnaires were administered and therefore, the results must be interpreted with caution because of recall bias or over- or understatement of experiences and/or symptoms. Within studies regarding traumatic experiences, there is always a potential for bias due to distorted answers for reasons of minimization [90]. Another limitation that must be considered is the generally small sample size in comparison to studies conducted in adults. In addition, the sample at hand is rather specific and therefore generalizability may be limited. Furthermore, there is an uneven gender distribution in the sample, which may have impacted the comparison between groups. As there is a gender difference between the classes, this fact must be included in the interpretation of the results. In addition, the data must be interpreted with caution because there was a rather large amount of missing data regarding protective factors. Lastly, the assessment of adaptation at one specific point may be limited, since resilience is a dynamic, constantly changing process [4]. Therefore, longitudinal investigations of adaptation are needed.

### Implications

A focus on resilience and its promotion is necessary given the detrimental consequences of traumatic experiences and their cost for the individual and the society [1]. Since resilience may show its manifestation differently in distinct populations at risk [27, 33], it is vital that specific populations are examined thoroughly to implement appropriate strategies and tailored interventions to mitigate suffering. The present results suggest that protective factors like caregiver and peer support, self-efficacy and SOC might promote healthy adaptation in the face of adversity in foster children and adolescents. For example, programs for children living in residential care could be implemented where they could connect with their peers, such as after-school activities and summer programs. There should also be adults in schools who can be trusted and to whom students can turn. To promote self-efficacy and SOC, it must be possible to have successful experiences, therefore, tasks that neither over- nor under-challenge the children are a good basis for this. As resilience research emphasizes the relevance of promoting protective factors as a basis for positive adaptation in the face of adversity [27], the present findings are vital to inform clinical psychology.

As was shown in the present study, gender plays an important role in the examination of resilience. Since females and males differ both in prevalence rates of psychopathological symptoms [60, 62] and in different domains of adaptation such as delinquency [91], it is necessary to consider which indicators of resilience are evaluated. A focus on external conformity and functioning well in society may often hide underlying problems. Furthermore, gender must always be considered in any study of resilience to control for any biases caused by included indicators of resilience. In addition, gender differences were also reported considering traumatic experiences. This should also be taken into account in further research.

## Supplementary Information

**Additional file 1. **Cut-offscores for the measures used in the study.

## Data Availability

The present data were assessed in the course of a research project commissioned and funded by the government of Lower Austria. Therefore, data cannot be shared due to legal reasons. However, can be available from the corresponding author upon reasonable request.
