# Supplementary material for: Resilience in the face of adversity: classes of positive adaptation in trauma-exposed children and adolescents in residential care
Source: BMC Psychol. 2023 Jan 30;11:30. doi: 10.1186/s40359-023-01049-x (PMC9887823; doi:10.1186/s40359-023-01049-x)
Supplement: Supplementary file 1 — Additional file 1. Cut-offscores for the measures used in the study. [file 40359_2023_1049_MOESM1_ESM.docx]

**Supplement 1. Cut-off scores for the measures used in the study**

1. *The Childhood Trauma Questionnaire (CTQ)* was used to assess five different types of childhood maltreatment. Each item is rated on a five-point Likert scale, resulting in a score ranging from 5 to 25. Based on this, the severity of the individual scales can be divided into "*Not to minimal*", "*Low to moderate*", "*Moderate to severe*" and "*Severe to extreme*". Since the calculation of the prevalence of maltreatment includes the severities "Moderate to severe" and "Severe to extreme"[92], the dichotomy was based on this. For the individual scales, the cut-off values were as follows: emotional abuse (cut-off = 13), physical abuse (cut-off = 10), sexual abuse (cut-off = 8), emotional neglect (cut-off = 15), and physical neglect (cut-off = 10).
2. *The Child and Adolescent Trauma Screen (CATS)* was used to dichotomously (*‘Yes’/’No’*) assess 15 potentially traumatic events. The sum score was used for calculations.
3. *International Trauma Questionnaire- Child and Adolescent Version (ITQ-CA):* assessment of PTSD and CPTSD. Diagnostic algorithms are as follows:

A diagnosis of PTSD requires the endorsement of one of two symptoms from the symptom clusters of (1) reexperiencing in the here and now, (2) avoidance, and (3) sense of current threat, plus endorsement of at least one indicator of functional impairment associated with these symptoms. Endorsement of a symptom or functional impairment item is defined as a score > 2.

A diagnosis of CPTSD requires the endorsement of one of two symptoms from each of the three PTSD symptoms clusters (re-experiencing in the here and now, avoidance, and sense of current threat) and one of two symptoms from each of the three Disturbances in Self-Organization (DSO) clusters: (1) affective dysregulation, (2) negative self-concept, and (3) disturbances in relationships. Functional impairment must be identified where at least one indicator of functional impairment is endorsed related to the PTSD symptoms and one indicator of functional impairment is endorsed related to the DSO symptoms. Endorsement of a symptom or functional impairment item is defined as a score > 2. An individual can receive either a diagnosis of PTSD or CPTSD, not both. If a person meets the criteria for CPTSD, that person does not also receive a PTSD diagnosis.

See:<https://www.traumameasuresglobal.com/_files/ugd/be25b4_f2d205d2cde4448aaedbfb7cda78ca35.pdf>

1. *Child Behavior Checklist, Youth Self Report* *(CBCL, YSR 11-18R):* assessment of internalizing problems, externalizing problems, interpersonal problems and thought problems. Cut-offs for the respective scales are as follows: For the scales internalizing problems and externalizing problems, the cut-off was at a T-value of ≥60. For the scales interpersonal problems and thought problems, the cut-off was at a T-value of ≥65.
2. *Adolescent Dissociative Experiences Scale-8 (ADES-8)*: assessment of dissociation. This questionnaire consists of eight items on a numerical scale ranging from one to ten. The cut-off score is 3. This score is computed based on the sum score of the questions divided by the number of questions.

See: [**https://www.kindertraumainstitut.de/de/Materialien/;40-Screening-Instrumente_fuer_dissoziative_Stoerungen_II_%28A-DES_II%29**](https://www.kindertraumainstitut.de/de/Materialien/;40-Screening-Instrumente_fuer_dissoziative_Stoerungen_II_%28A-DES_II%29)

1. *Questionnaire for Resources in Children and Adolescents (FRKJ, Fragebogen für Ressourcen im Kindes- und Jugendalter)*: assessment of protective factors self-efficacy, SOC, peer support and caregiver support. Higher values indicate better resources. For the cut-offs see supplementary table 1.

Supplementary table 1

Stanine, PR values and corresponding classification for the FRKJ questionnaire.

| Stanine | PR | Classification |
| --- | --- | --- |
| 1 | 0-4 | Far below average |
| 2 | 5-11 | Below average |
| 3-7 | 12-89 | Average |
| 8 | 90-96 | Above average |
| 9 | 97-100 | Far above average |

*Note.* Stanine = Stanine values; PR = percentile rank.
